# Single Amino Acid Change in STING Leads to Constitutive Active Signaling

**DOI:** 10.1371/journal.pone.0120090

**Published:** 2015-03-19

**Authors:** Eric D. Tang, Cun-Yu Wang

**Affiliations:** Laboratory of Molecular Signaling, Division of Oral Biology & Medicine, UCLA School of Dentistry, Los Angeles, CA, United States of America; University of Colorado School of Medicine, UNITED STATES

## Abstract

The production of cytokines by the immune system in response to cytosolic DNA plays an important role in host defense, autoimmune disease, and cancer immunogenicity. Recently a cytosolic DNA signaling pathway that is dependent on the endoplasmic reticulum adaptor and cyclic dinucleotide sensor protein STING has been identified. Association of cytosolic DNA with cyclic-GMP-AMP synthase (cGAS) activates its enzymatic activity to synthesize the cyclic dinucleotide second messenger cGAMP from GTP and ATP. Direct detection of cGAMP by STING triggers the activation of IRF3 and NF-kB, and the production of type I interferons and proinflammatory cytokines. The mechanism of how STING is able to mediate downstream signaling remains incompletely understood although it has been shown that dimerization is a prerequisite. Here, we identify a single amino acid change in STING that confers constitutive active signaling. This mutation appears to both enhance ability of STING to both dimerize and associate with its downstream target TBK1.

## Introduction

Despite the fact that DNA has long been known to trigger immune responses, only within the past few years have the underlying molecular mechanisms been significantly uncovered. Recently, important progress has been made in the elucidation of a cytosolic DNA signaling pathway involving the stimulator of IFN genes (STING), an endoplasmic reticulum membrane adaptor protein which functions as a sensor for cyclic dinucleotides (CDNs) [1–6]. In this pathway, cytosolic DNA induces the production of type I interferons (IFNs) and other cytokines in a STING-dependent manner [7]. Several DNA sensors have been proposed to function in this signaling pathway, but only one, cyclic-GMP-AMP (cGAMP) synthase (cGAS), appears to be required for DNA-mediated immune responses regardless of cell type [8]. Binding of DNA to cGAS induces its dimerization and a conformational change that opens up its catalytic pocket, thereby activating the synthesis of the CDN 2’3’-cGAMP from ATP and GTP [9–13]. cGAMP serves as a secondary messenger to activate STING-dependent signaling by binding to STING and inducing a conformational change, which allows for the activation of the transcription factors IRF3 and NF-kB through the kinases TBK1 and IKK, respectively [2,5,6]. It has been demonstrated that dimerization of STING is induced in response to cytosolic DNA and is important for STING activation [5,14]. Crystal structures of CDNs complexed with the STING dimer interface suggest that binding of CDNs to STING may stabilize its dimeric active configuration [15].

The cGAS-STING cytosolic DNA signaling pathway is likely to be tightly regulated, as conditions that promote excess cytosolic DNA in mice can lead to autoimmune disease that is dependent on STING [16,17]. Also, gain-of-function point mutations in human STING have been recently found to be associated with autoinflammatory disease [18]. Thus, overactivation of STING-dependent signaling can lead to dysregulation of the production of cytokines, which may in turn contribute to autoimmune and autoinflammatory disease. On the other end of the spectrum, loss of STING function in mice leads to an increased susceptibility to infection by DNA viruses such as herpes simplex virus 1 (HSV1), vaccinia virus (VACV), and murine gammaherpesvirus 68 (MHV68) [19,20]. Retroviruses such as HIV, which use reverse-transcribed DNA to propagate, also appear to use the cGAS-STING pathway to induce innate immune system activation [21]. Thus, proper regulation of STING-mediated signaling appears to be critical for the prevention of infectious disease and immune dysregulation in mice and humans. Recently, STING has also been implicated in tumor immunogenicity by mediating the induction of type I IFNs in dendritic cells in response to tumor DNA. The identification and characterization of gain-of-function STING mutants will be useful in understanding the regulatory mechanisms of STING-dependent signaling. In this report, we reveal a single amino acid change in STING that leads to its constitutive activation of downstream signaling, by apparently increasing its propensity to dimerize and associate with TBK1.

## Materials and Methods

### Cell culture and transfections

HEK293 and HEK293T cells were from ATCC and were grown in Dulbecco’s modified Eagle’s medium (DMEM) supplemented with 10% fetal bovine serum, 50 units penicillin/ml, and 50 mg streptomycin/ml (Life Technologies). Transient transfections were using Lipofectamine 2000 (Life Technologies) and luciferase assays were performed as previously described using Renilla as an internal control [22]. For cGAMP treatments, 1ug of cGAMP was complexed with 1ul of Lipofectamine 2000 (Life Technologies) in 100ul total Opti-MEM (Life Technologies) and added to HEK293T cells seeded in 500ul of growth media in 24 well tissue culture plates (Falcon). Samples for all luciferase assays were performed in duplicate and results shown are representative of three independent experiments.

### Reagents and plasmids

STING and TBK1 rabbit polyclonal antibodies were from Cell Signaling Technology. FLAG M2 and α-tubulin monoclonal mouse antibodies were from Sigma. Expression constructs for STING were constructed by PCR amplification of the STING cDNA from a full-length cDNA purchased from Open Biosystems (RefSeq: NM_198282) and cloning into the BamHI and EcoRI sites in pcDNA3. The 5’ end primer included a BamHI recognition and Kozak consensus sequence prior to the initiation methionine (5’-CGGGATCCGCCACCATGCCCCACTCCAGCCT-3’). The 3’ end primer included an EcoRI recognition sequence and was designed to introduce an AU1 epitope tag to the C-terminus of STING (5’-GGAATTCAGATGTAGCGGTATGTGTCAGAGAAATCCGTGCGGAG-3’). Codon 132 was mutated to encode arginine instead of histidine as Arg 132 represents the most prevalent haplotype [23]. Missense mutations in the *TMEM173* gene found in cancer tissues and cell lines were identified by searching the Catalogue of Somatic Mutations in Cancer (COSMIC) database (cancer.sanger.ac.uk). All mutations in STING were introduced by using the Quikchange Site-Directed PCR Mutagenesis Kit (Agilent Technologies). Lentiviral STING expression constructs were constructed by PCR amplification of wild-type and mutant STING cDNAs and replacement of the LAMP1-mRFP-FLAG insert in pLJM1 (Addgene #34611) between NheI and EcoRI recognition sites. An empty control lentiviral construct was created by cleavage of pLKO-puro FLAG SREBP1 (Addgene #32017) with EcoRI and self-ligation. The IFNβ promoter luciferase constructs pLUC-IFNβ, pLUC-PRD(III-I)_3,_ and pLUC-PRD(II)_2_ have been described previously [24]. The mammalian expression plasmid for full-length cGAS, pCMV-SPORT6-cGAS, was purchased from Open Biosystems (Clone ID# 6015929). The form of cGAMP with a 2’-5’ phosphodiester linkage between the guanosine and the adenosine (2’3’-cGAMP, InvivoGen) that has been shown to be produced by cGAS was used in stimulation experiments.

### Immunoblotting and coimmunoprecipitations

For preparation of whole cell lysates, cells were lysed in Triton X-100 lysis buffer (20 mM Tris-HCl, pH 7.5, 150 mM NaCl, 1% Triton X-100, 1 mM EDTA, 30 mM NaF, 2 mM sodium pyrophosphate, 0.1 mM Na_3_VO_4_, 10mM β-glycerophosphate. 1 mM dithiothreitol) supplemented with complete EDTA-free protease inhibitor cocktail (Agilent Technologies). For coimmunoprecipitation experiments, cells were lysed in CHAPS lysis buffer (40mM Hepes pH 7.4, 2mM EDTA, 10mM sodium pyrophosphate, 0.3% CHAPS, 50mM NaF, 10mM β-glycerophosphate. 1 mM dithiothreitol) supplemented with complete EDTA-free protease inhibitor cocktail (Agilent Technologies). Lysates were incubated with FLAG M2 at 4°C for 1hr. 7ul of 1:1 protein G sepharose slurry (Agilent Technologies) was added and lysates were incubated at 4°C for an additional hr. All proteins were separated by SDS-PAGE and probed using ECL western blotting.

### IFNβ measurements and flow cytometry

Human IFNβ enzyme-linked immunosorbent assays were performed according to the manufacturer’s instructions (PBL). Briefly, 36 h following plasmid transfection of HEK293 cells with pcDNA3 or STING expression plasmid, supernatants were collected and analyzed. Vesicular stomatitis virus-green fluorescent protein (VSV-GFP) infection measurements were performed as described previously [22]. Cells were analyzed for GFP fluorescence by flow cytometry 12 h following infection (UCLA Flow Cytometry Core).

## Results and Discussion

**Fig 1:**
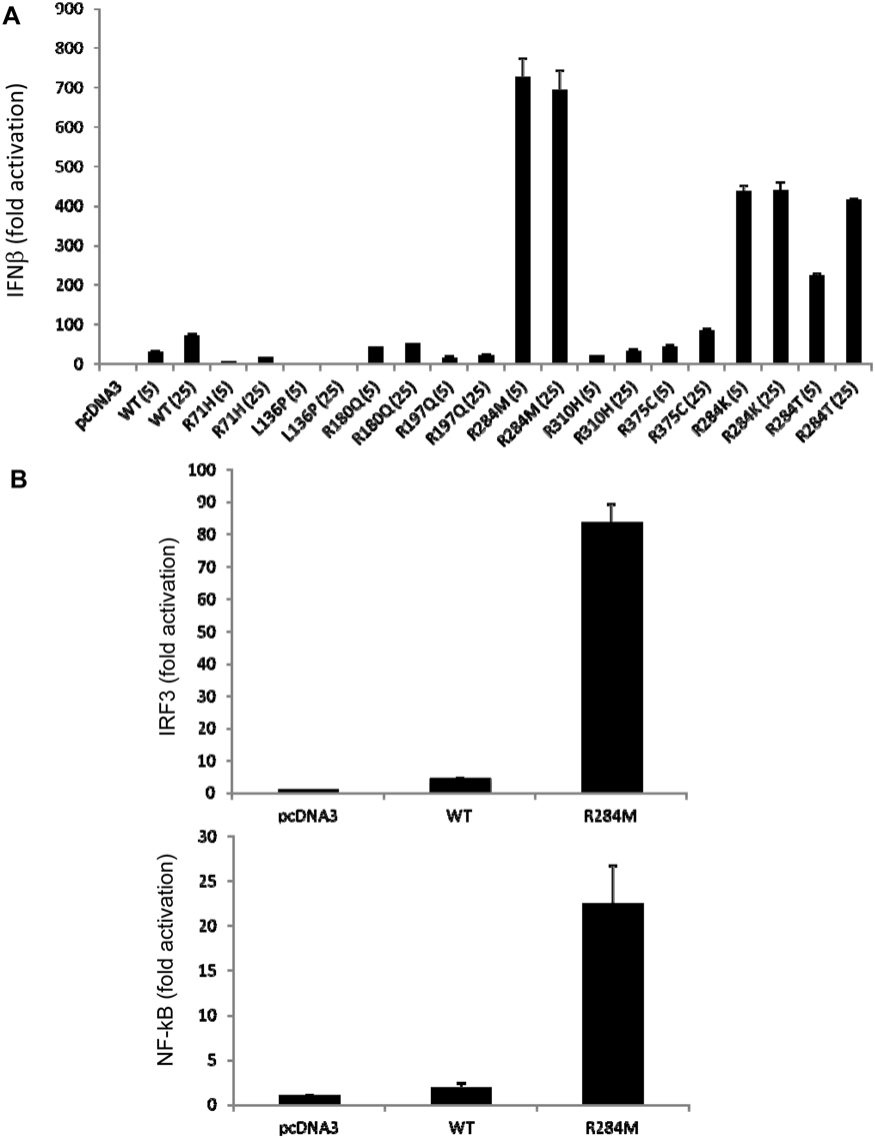
Effect of missense mutations in STING found in cancer cells on activation of the IFNβ promoter. (A) Luciferase reporter assay for HEK293T cells cotransfected with pcDNA3, wild-type STING, or STING missense mutants, and an IFNββ promoter reporter construct, pLUC- IFNβ. 5 ng (5) or 25 ng (25) of STING expression construct was transfected as indicated. The normalized relative luciferase activity activity is shown (mean +/- standard deviation) (B) Luciferase reporter assay for HEK293T cells cotransfected with pcDNA3, wild-type STING, or R284M mutant, and the pLUC-PRD(III-I)_3_ or pLUC-PRD(II)_2_ reporter construct as indicated.

**Fig 2:**
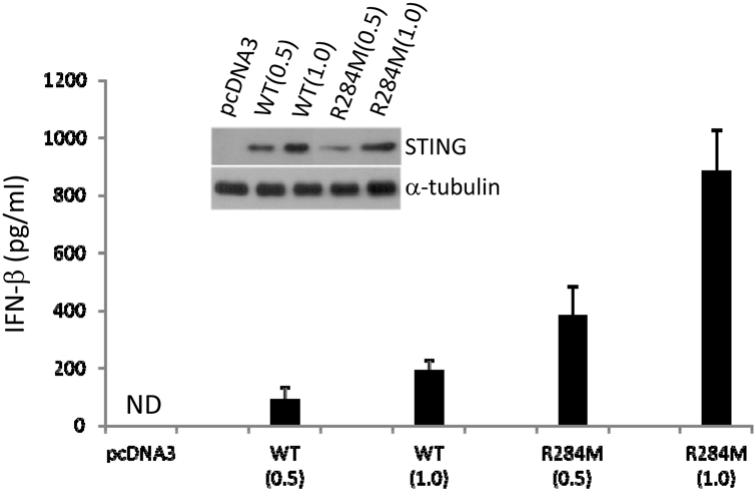
Induction of type I IFN production by the R284M mutation. Human IFNβ enzyme-linked immunosorbent assays at 36 h with tissue culture supernatants of HEK293 cells transfected with pcDNA3, wild-type STING, or STING R284M. Samples were prepared in duplicate. Either 0.5ug or 1.0ug of plasmid was transfected as indicated. The concentration of IFNβ is shown (mean +/- standard deviation). ND, not detectable. Expression of STING and α-tubulin proteins as determined by immunoblot from cell lysates is shown in the insert.

HEK293T cells do not express detectable endogenous STING or cGAS protein, but ectopic STING expression can enhance IFNβ promoter activation and render cells responsive to CDN treatment [2,8,25]. We used an IFNβ promoter luciferase assay to assess the effect of various STING missense mutations found in human cancer tissues and cell lines, as identified from searching the COSMIC database (see Materials and Methods). We created expression plasmids for wild-type human STING cDNA and various missense mutants. Each inserted cDNA was designed to contain a codon for arginine at position 232. The Arg 232 allele was previously found to be prevalent upon extensive analysis of DNA sequences from human populations, and thus should be considered wild-type STING [23]. Upon transfection of expression plasmids for wild-type and various mutant STING proteins, we found that several mutant STING proteins were impaired when compared to wild-type in inducing IFNβ promoter activation. However one of the mutants, containing a substitution of arginine 284 to methionine (R284M), was able to induce luciferase reporter activation to a significantly higher extent than wild-type STING (Fig. 1A). This R284M mutant displayed an enhanced ability to activate luciferase reporter constructs with isolated binding sites for IRF3 or NF-kB, demonstrating that both transcription factors were activated (Fig. 1B). Alternative substitution of arginine 284 with lysine or threonine (R284K and R284T) also resulted in hyperactive mutants, suggesting that multiple types of amino acids placed at position 284 can confer hyperactivity (Fig. 1A). Thus, the R284M mutant possesses a significantly enhanced ability to activate IRF3 and NF-kB, and the IFNβ promoter. To determine whether the R284M mutant could also induce IFNβ protein production, we transfected wild-type STING or the R284M mutant into HEK293 cells and measured IFNβ levels in tissue culture supernatants. In accordance with our luciferase reporter assay results, we found that the R284M mutant was significantly more potent than wild-type STING in the production of IFNβ protein as well (Fig. 2).

**Fig 3:**
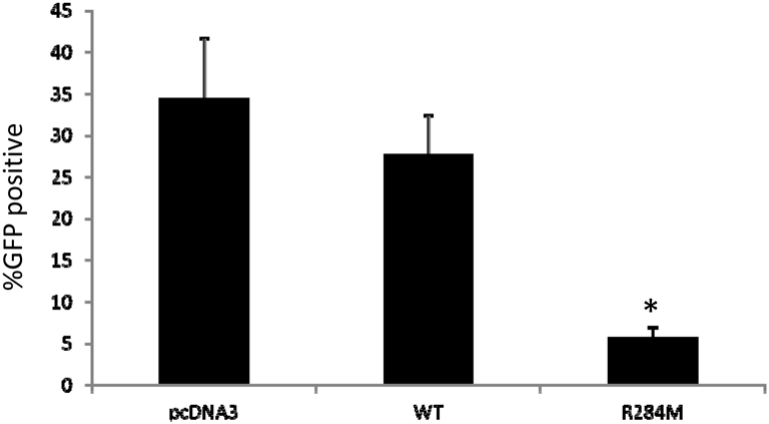
Protection of cells from VSV infection by the R284M mutant. Monitoring for VSV-GFP replication by flow cytometry with infected HEK293 cells transiently transfected with pcDNA3, wild-type STING, or R284M mutant. The percentage of cells positive for GFP is indicated (mean +/- standard deviation). Samples were performed in triplicate. *, *P* value of < 0.005 for comparison with pcDNA3 (n = 3).

Type I interferons such as IFNβ can potently inhibit viral replication. Given that the R284M mutant could induce IFNβ production, we tested this STING mutant for antiviral activity as well. We transfected HEK293 cells with wild-type STING or the R284M mutant and subsequently infected cells with recombinant VSV harboring a green fluorescent protein transgene (VSV-GFP). Following infection, we determined the percentage of infected cells by flow cytometry. We found that the percentage of infected cells was significantly diminished in cells transfected with the R284M mutant while wild-type STING had a much lesser effect (Fig. 3). These results suggest that the R284M mutant can function as a potent inducer of type I interferon and an inhibitor of viral replication.

**Fig 4:**
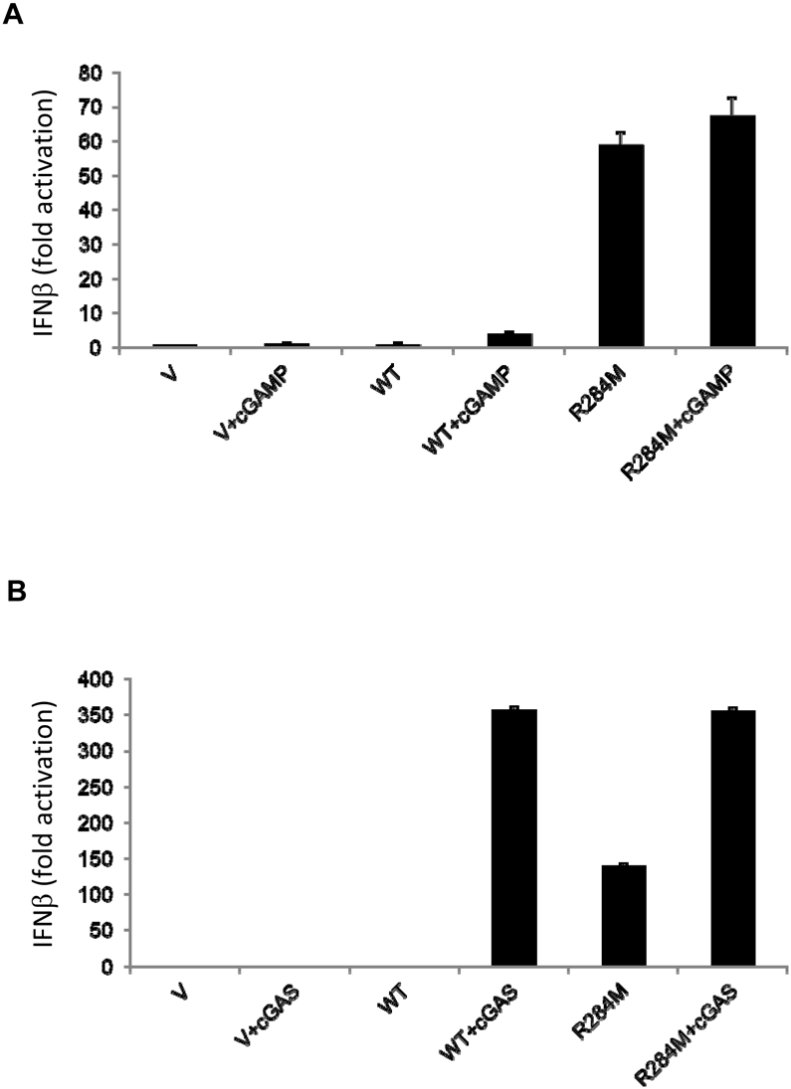
The R284M mutation triggers constitutively active STING-dependent signaling. (A) Responsiveness of HEK293T cells with stable STING expression to cGAMP. Luciferase reporter assay for HEK293T cells stably expressing empty vector (V), wild-type STING (WT), or STING R284M. All cells were transfected with an IFNβ promoter reporter construct, pLUC- IFNββββ 4 hrs after transfection, new growth media was added and cells were either mock transfected or transfected with 0.5ug/ml cGAMP as indicated. Luciferase activity was measured 16hrs later and analyzed as in Fig. 1. (B) Responsiveness of HEK293T cells with stable STING expression to exogenous cGAS. Luciferase reporter assay for HEK293T cells stably expressing empty vector (V), wild-type STING (WT), or STING R284M. Cells were transfected with pcDNA3 (V) or pCMV-SPORT6-cGAS (cGAS) as indicated. All cells were transfected with an IFNβ promoter reporter construct, pLUC- IFNβ. Luciferase activity was measured 20 hrs following transfection.

To examine whether the R284M mutant conferred on STING constitutive active signaling, we created pools of 293T cells stably expressing wild-type STING or the R284M mutant. These cells were compared in IFNβ promoter luciferase assays in the presence or absence of cGAMP or cGAS cotransfection. We found that vector control cells were not sensitive to treatment with cGAMP or expression of cGAS, as expected, due to the absence of STING (Fig. 4). However, wild-type STING stable expression was able to confer on 293T cells IFNβ promoter activation only in the presence of cGAMP or cGAS transfection, consistent with previous findings [25,26]. In contrast, cells with stable expression of the R284M mutant displayed robust IFNβ promoter activation in the presence or absence of cGAMP or cGAS (Fig. 4). These results indicate that the R284M mutation renders STING-dependent signaling constitutively active.

**Fig 5:**
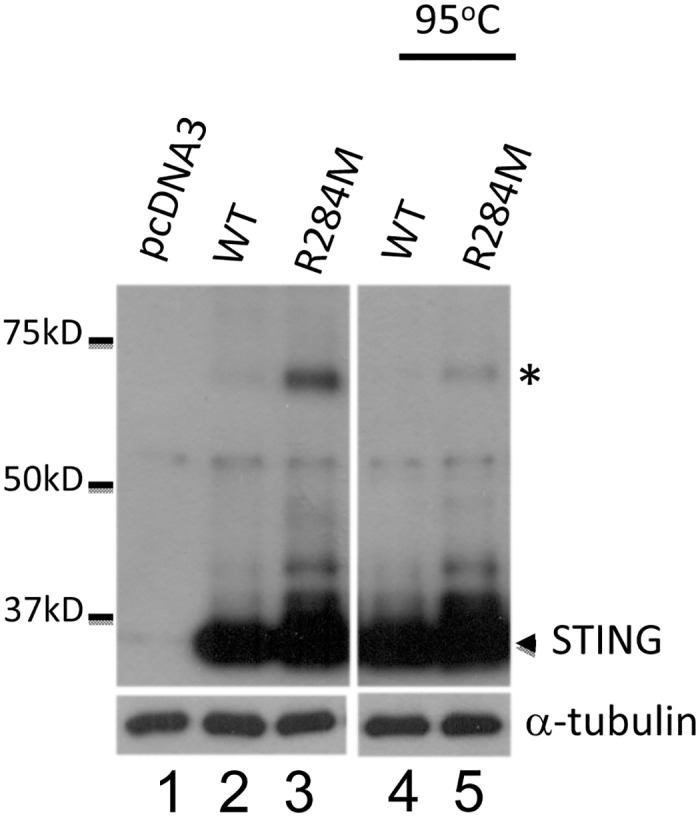
STING with an R284M mutation forms SDS-resistant dimers. Immunoblot of whole cell lysates from HEK293T cells transfected with wild-type STING or the R284M mutant analyzed by SDS-PAGE using a STING rabbit polyclonal antibody (Cell Signaling). Samples were heated at 95°C for 5 min as indicated (lanes 4 and 5) prior to loading. Positions of protein molecular weight markers are indicated on left-hand side. The arrowhead indicates the 35-kDa STING monomer and the asterisks indicates the 70-kDa presumptive STING dimer. For a loading control, α-tubulin antibody was used.

Dimerization of STING has been previously shown to occur following the introduction of cytosolic DNA and to be critical for STING-dependent signal transduction [5,14]. Upon analysis of cell lysates from cells expressing wild-type STING and the R284M mutant by SDS-PAGE and immunoblotting, we found that the R284M mutant displayed a band of predicted molecular weight 35kD along with a slower mobility band corresponding with a molecular weight of 70kD, consistent with a dimeric species of STING (Fig. 5). This dimeric species was not prominent with wild-type STING and was disrupted by heating samples at 95°C. This result suggests that the R284M mutant has an enhanced ability to form SDS-resistant dimers, which may be related to its elevated IFNβ inducing activity.

**Fig 6:**
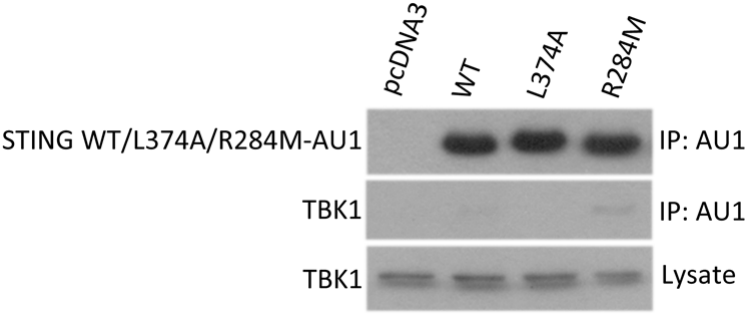
Enhanced association of STING with an R284M mutation with TBK1. Immunoblot of anti-AU1 immunoprecipitates and whole cell lysates from HEK293T cells transfected with AU1-tagged wild-type STING, L374A, or R284M mutant analyzed by SDS-PAGE. Proteins were probed using an AU1 mouse monoclonal or TBK1 rabbit polyclonal antibody as indicated. Cells were lysed 20 hrs following transfection.

One possible mechanism whereby the R284M mutant may be constitutively active may be through altered binding to downstream target proteins. Previously, STING was been shown to associate its downstream target kinase, TBK1, and this association was found to be enhanced upon CDN binding [5,14]. In order to examine the ability of the R284M mutant to bind TBK1, we performed coimmunoprecipitation experiments in HEK293T cells transfected with wild-type STING or the R284M mutant. We found that wild-type STING was able to weakly pull down endogenous TBK1 (Fig. 6). In contrast, the R284M mutant was able to coimmunoprecipitate significantly more TBK1. We also tested a previously characterized inactive mutant of STING, L374A, which is completely inactive in inducing IRF3 activation and IFNβ production [27]. This mutant displayed no detectable binding to TBK1. Thus, the R284M mutant has an enhanced ability to bind to TBK1, and this difference provides a possible explanation for its constitutive active state.

Dimerization of STING has been previously shown to be essential for IFNβ induction in response to cytosolic DNA sensing. Our results suggest that the R284M mutation may trigger constitutive active STING-dependent signaling by facilitating STING dimerization and/or TBK1 binding and activation. Although dimerization and TBK1 binding both are correlated with activation of STING-dependent signaling, it remains to be seen whether TBK1 association is dependent on STING dimerization as a previous report found no defect in binding of two dimerization impaired STING mutants to TBK1. Interestingly, Arg 284 is located in the STING C-terminal tail, which is predicted to lie in the lumen of the endoplasmic reticulum, separate from the aa 153–177 region that has previously been shown to participate in dimerization [5,14]. Given that consititutive active STING-dependent signaling can be triggered by multiple types of residues substituted at Arg 284, this residue appears unlikely to be involved directly in dimerization interactions. Instead, we propose that mutation of Arg 284 may promote or inhibit binding of a cellular factor that stabilizes or impairs STING dimerization, respectively. For instance, it is possible that the R284M mutation interferes with binding of a negative regulatory protein that interferes with dimerization. The recently discovered STING binding partner and negative regulator, NLRC3, is a possible such candidate protein [28]. Identification of the molecular mechanism of how the R284M mutant renders STING constitutively active is likely to reveal insights into how STING-dependent signaling is regulated. Also, these studies may be helpful in guiding the construction of biological agents that can enhance the immune response to vaccines or to promote host defense.

## References

[1] Burdette DL, Monroe KM, Sotelo-Troha K, Iwig JS, Eckert B, Hyodo M, et al STING is a direct innate immune sensor of cyclic di-GMP. Nature. 2011; 478: 515–518. 10.1038/nature10429 21947006 PMC3203314

[2] Ishikawa H, Barber GN STING is an endoplasmic reticulum adaptor that facilitates innate immune signalling. Nature. 2008; 455: 674–678. 10.1038/nature07317 18724357 PMC2804933

[3] Jin L, Hill KK, Filak H, Mogan J, Knowles H, Zhang B, et al MPYS is required for IFN response factor 3 activation and type I IFN production in the response of cultured phagocytes to bacterial second messengers cyclic-di-AMP and cyclic-di-GMP. J Immunol. 2011; 187: 2595–2601. 10.4049/jimmunol.1100088 21813776 PMC3159690

[4] Jin L, Waterman PM, Jonscher KR, Short CM, Reisdorph NA, Cambier JC MPYS, a novel membrane tetraspanner, is associated with major histocompatibility complex class II and mediates transduction of apoptotic signals. Mol Cell Biol. 2008; 28: 5014–5026. 10.1128/MCB.00640-08 18559423 PMC2519703

[5] Sun W, Li Y, Chen L, Chen H, You F, Zhou X, et al ERIS, an endoplasmic reticulum IFN stimulator, activates innate immune signaling through dimerization. Proc Natl Acad Sci U S A. 2009; 106: 8653–8658. 10.1073/pnas.0900850106 19433799 PMC2689030

[6] Zhong B, Yang Y, Li S, Wang YY, Li Y, Diao F, et al The adaptor protein MITA links virus-sensing receptors to IRF3 transcription factor activation. Immunity. 2008; 29: 538–550. 10.1016/j.immuni.2008.09.003 18818105

[7] Cai X, Chiu YH, Chen ZJ The cGAS-cGAMP-STING pathway of cytosolic DNA sensing and signaling. Mol Cell. 2014; 54: 289–296. 10.1016/j.molcel.2014.03.040 24766893

[8] Sun L, Wu J, Du F, Chen X, Chen ZJ Cyclic GMP-AMP synthase is a cytosolic DNA sensor that activates the type I interferon pathway. Science. 2013; 339: 786–791. 10.1126/science.1232458 23258413 PMC3863629

[9] Civril F, Deimling T, de Oliveira Mann CC, Ablasser A, Moldt M, Witte G, et al Structural mechanism of cytosolic DNA sensing by cGAS. Nature. 2013; 498: 332–337. 10.1038/nature12305 23722159 PMC3768140

[10] Gao P, Ascano M, Wu Y, Barchet W, Gaffney BL, Zillinger T, et al Cyclic [G(2',5')pA(3',5')p] is the metazoan second messenger produced by DNA-activated cyclic GMP-AMP synthase. Cell. 2013; 153: 1094–1107. 10.1016/j.cell.2013.04.046 23647843 PMC4382009

[11] Kranzusch PJ, Lee AS, Berger JM, Doudna JA Structure of human cGAS reveals a conserved family of second-messenger enzymes in innate immunity. Cell Rep. 2013; 3: 1362–1368. 10.1016/j.celrep.2013.05.008 23707061 PMC3800681

[12] Li X, Shu C, Yi G, Chaton CT, Shelton CL, Diao J, et al Cyclic GMP-AMP synthase is activated by double-stranded DNA-induced oligomerization. Immunity. 2013; 39: 1019–1031. 10.1016/j.immuni.2013.10.019 24332030 PMC3886715

[13] Zhang X, Wu J, Du F, Xu H, Sun L, Chen Z, et al The cytosolic DNA sensor cGAS forms an oligomeric complex with DNA and undergoes switch-like conformational changes in the activation loop. Cell Rep. 2014; 6: 421–430. 10.1016/j.celrep.2014.01.003 24462292 PMC3969844

[14] Ouyang S, Song X, Wang Y, Ru H, Shaw N, Jiang Y, et al Structural analysis of the STING adaptor protein reveals a hydrophobic dimer interface and mode of cyclic di-GMP binding. Immunity. 2012; 36: 1073–1086. 10.1016/j.immuni.2012.03.019 22579474 PMC3654694

[15] Huang YH, Liu XY, Du XX, Jiang ZF, Su XD The structural basis for the sensing and binding of cyclic di-GMP by STING. Nat Struct Mol Biol. 2012; 19: 728–730. 10.1038/nsmb.2333 22728659

[16] Ahn J, Gutman D, Saijo S, Barber GN STING manifests self DNA-dependent inflammatory disease. Proc Natl Acad Sci U S A. 2012; 109: 19386–19391. 10.1073/pnas.1215006109 23132945 PMC3511090

[17] Gall A, Treuting P, Elkon KB, Loo YM, Gale M Jr., Barber GN, et al Autoimmunity initiates in nonhematopoietic cells and progresses via lymphocytes in an interferon-dependent autoimmune disease. Immunity. 2012; 36: 120–131. 10.1016/j.immuni.2011.11.018 22284419 PMC3269499

[18] Liu Y, Jesus AA, Marrero B, Yang D, Ramsey SE, Sanchez M, et al Activated STING in a Vascular and Pulmonary Syndrome. N Engl J Med. 2014; 371: 507–518. 10.1056/NEJMoa1312625 25029335 PMC4174543

[19] Li XD, Wu J, Gao D, Wang H, Sun L, Chen ZJ Pivotal roles of cGAS-cGAMP signaling in antiviral defense and immune adjuvant effects. Science. 2013; 341: 1390–1394. 10.1126/science.1244040 23989956 PMC3863637

[20] Schoggins JW, MacDuff DA, Imanaka N, Gainey MD, Shrestha B, Eitson JL, et al Pan-viral specificity of IFN-induced genes reveals new roles for cGAS in innate immunity. Nature. 2014; 505: 691–695. 10.1038/nature12862 24284630 PMC4077721

[21] Gao D, Wu J, Wu YT, Du F, Aroh C, Yan N, et al Cyclic GMP-AMP synthase is an innate immune sensor of HIV and other retroviruses. Science. 2013; 341: 903–906. 10.1126/science.1240933 23929945 PMC3860819

[22] Tang ED, Wang CY MAVS self-association mediates antiviral innate immune signaling. J Virol. 2009; 83: 3420–3428. 10.1128/JVI.02623-08 19193783 PMC2663242

[23] Jin L, Xu LG, Yang IV, Davidson EJ, Schwartz DA, Wurfel MM, et al Identification and characterization of a loss-of-function human MPYS variant. Genes Immun. 2011; 12: 263–269. 10.1038/gene.2010.75 21248775 PMC3107388

[24] Fitzgerald KA, McWhirter SM, Faia KL, Rowe DC, Latz E, Golenbock DT, et al IKKepsilon and TBK1 are essential components of the IRF3 signaling pathway. Nat Immunol. 2003; 4: 491–496. 12692549 10.1038/ni921

[25] Diner EJ, Burdette DL, Wilson SC, Monroe KM, Kellenberger CA, Hyodo M, et al The innate immune DNA sensor cGAS produces a noncanonical cyclic dinucleotide that activates human STING. Cell Rep. 2013; 3: 1355–1361. 10.1016/j.celrep.2013.05.009 23707065 PMC3706192

[26] Zhang X, Shi H, Wu J, Zhang X, Sun L, Chen C, et al Cyclic GMP-AMP containing mixed phosphodiester linkages is an endogenous high-affinity ligand for STING. Mol Cell. 2013; 51: 226–235. 10.1016/j.molcel.2013.05.022 23747010 PMC3808999

[27] Tanaka Y, Chen ZJ STING specifies IRF3 phosphorylation by TBK1 in the cytosolic DNA signaling pathway. Sci Signal. 2012; 5: ra20 10.1126/scisignal.2002521 22394562 PMC3549669

[28] Zhang L, Mo J, Swanson KV, Wen H, Petrucelli A, Gregory SM, et al NLRC3, a member of the NLR family of proteins, is a negative regulator of innate immune signaling induced by the DNA sensor STING. Immunity. 2014; 40: 329–341. 10.1016/j.immuni.2014.01.010 24560620 PMC4011014

